# Time-lapse reveals that osteoclasts can move across the bone surface while resorbing

**DOI:** 10.1242/jcs.202036

**Published:** 2017-06-15

**Authors:** Kent Søe, Jean-Marie Delaissé

**Affiliations:** Department of Clinical Cell Biology, Vejle Hospital/Lillebaelt Hospital, Institute of Regional Health Research, University of Southern Denmark, 7100 Vejle, Denmark

**Keywords:** Osteoclast, Bone resorption, Pit resorption, Trench resorption, Intermittent resorption, Continuous resorption

## Abstract

Bone erosion both demands that the osteoclast resorbs bone matrix and moves over the bone surface. It is widely accepted that these two activities alternate, because they are considered mutually exclusive since resorption is believed to involve an immobilizing seal to the bone surface. However, clear real-time observations are still lacking. Herein, we used specific markers and time-lapse to monitor live the spatiotemporal generation of resorption events by osteoclasts cultured on bone slices. In accordance with the current view, we found alternating episodes of resorption and migration resulting in the formation of clusters of round pits. However, very importantly, we also demonstrate that more than half of the osteoclasts moved laterally, displacing their extracellular bone-resorbing compartment over the bone surface without disassembling and reconstructing it, thereby generating long trenches. Compared to pit events, trench events show properties enabling higher aggressiveness: long duration (days), high erosion speed (two times faster) and long-distance erosion (several 100 µm). Simultaneous resorption and migration reflect a unique situation where epithelial/secretory and mesenchymal/migratory characteristics are integrated into just one cell phenotype, and deserves attention in future research.

## Abstract (precis)

**Summary:** Bone erosion requires that osteoclasts both resorb and migrate. According to common belief, these activities are mutually exclusive and alternate. Paradoxically, we show here simultaneous resorption and migration.

## INTRODUCTION

A functional osteoclast (OC) must be able to resorb bone matrix, but also to move its resorptive activity over the bone surface (Parfitt, 1993). This joined requirement is increasingly recognized as critical for proper bone modeling and remodeling (Novack and Faccio, 2011). Its cellular mechanism is currently ascribed to alternating resorption and cell spreading or cell migration episodes, and is referred to as the ‘resorption cycle’ model (Georgess et al., 2014; Lakkakorpi and Vaananen, 1991; Novack and Faccio, 2011; Saltel et al., 2004; Vives et al., 2011). These alternating episodes are in line with the fact that resorption and cell spreading/migration activities are considered mutually exclusive, as they depend on distinct cellular organizations that are reminiscent of epithelial and mesenchymal phenotypes, respectively (Lakkakorpi and Vaananen, 1991; Saltel et al., 2004; Takahashi et al., 2007). For resorption, the cytoskeletal configuration is like that found in a polarized secretory cell; a major feature of this phenotype is the organization of podosomes in a sealing zone (SZ) (Luxenburg et al., 2007; Saltel et al., 2004), which is believed to immobilize the OC and seal-off an extracellular bone resorption compartment; this SZ surrounds the ruffled border, which covers the resorption compartment and mediates secretion of resorption factors and uptake of resorption products. Spreading/migration requires a distinct cytoskeletal configuration where the ruffled border, the SZ and the resorption compartment are lost (Georgess et al., 2014; Vives et al., 2011), and the podosomes appear in clusters or rings (Georgess et al., 2014; Saltel et al., 2004). Thus, the resorption cycle model involves a constant coordination between construction and disassembly of ‘stationary’ resorption compartments, and repeated spreading or migratory episodes (Georgess et al., 2014; Lakkakorpi and Vaananen, 1991; Novack and Faccio, 2011; Saltel et al., 2004; Vives et al., 2011).

The main observation supporting the resorption cycle model is that OCs cultured on bone slices can lead to clusters or trails of round excavations defined as ‘pits’. This pattern is interpreted as successive resorption activities, each drilling perpendicular to the bone surface and separated by spreading/migration episodes (Georgess et al., 2014; Lakkakorpi and Vaananen, 1991; Saltel et al., 2004). The need for direct observations supporting this interpretation led to several attempts to follow bone-resorbing OCs in real time (Kanehisa and Heersche, 1988 and references therein; Taylor et al., 1989), but the lack of appropriate live imaging tools compromised clear-cut information. Meanwhile, the ‘resorption cycle’ has remained the only available model to explain how resorptive activity moves over the bone surface.

However, there are observations that question whether this commonly accepted resorption cycle model is really universal. Important in this respect, OCs do not only make round pits, but also long resorption cavities appearing as continuous ‘trenches’ (Boyde et al., 1986; Bruzzaniti et al., 2005; Dempster et al., 1986; Gentzsch et al., 2003; Jones et al., 1985; Merrild et al., 2015; Mulari et al., 2003; Nesbitt and Horton, 1997; Rumpler et al., 2013; Soe and Delaisse, 2010; Soe et al., 2013; Susa et al., 2004; Taylor et al., 1989; Zhuo et al., 2014). They are often referred to as tracks, trails or long pits. They have been interpreted as a series of contiguous pits according to the logics of the resorption cycle theory (Georgess et al., 2014; Novack and Faccio, 2011; Rumpler et al., 2013; Vives et al., 2011). However, their geometry and smooth appearance suggest that they could also be interpreted as being generated by OCs that persistently resorb laterally, moving across the bone surface (Merrild et al., 2015; Mulari et al., 2003; Nesbitt and Horton, 1997; Soe and Delaisse, 2010; Soe et al., 2013; Stenbeck and Horton, 2000). Furthermore, when observing trench-forming OCs from above, the SZ appears to be crescent-shaped instead of annular, as typical of pit-forming OCs (Merrild et al., 2015; Mulari et al., 2003; Nesbitt and Horton, 1997; Rumpler et al., 2013; Stenbeck and Horton, 2000; Taylor et al., 1989). This suggests a different orientation of the SZ and of the ruffled border it surrounds.

The present study takes advantage of recently developed fluorogenic probes allowing live imaging of actin (Lukinavicius et al., 2014). This new tool allowed us to clearly monitor the OC resorptive activity on bone slices through time-lapse, visualizing simultaneously (1) the SZ through actin staining, (2) removal of Rhodamine-labeled collagen, and (3) changes in refringency as observed by phase-contrast microscopy. Our study reveals for the first time how resorption events, whether pits or trenches, originate and progress in space and time. More importantly, it addresses a so far neglected hypothesis: that the OC can continuously move its resorption compartment over the bone surface and that this – rather than the resorption cycle – is the relevant mechanism explaining the generation of trenches (Merrild et al., 2015; Mulari et al., 2003; Nesbitt and Horton, 1997; Soe and Delaisse, 2010; Soe et al., 2013; Stenbeck and Horton, 2000). In order to understand how the resorption axis of trench-forming OCs can be lateral, we visualized the three-dimensional (3D) orientation of the SZ by using confocal microscopy. Importantly, the kinetics of pit and trench formation were also compared.

## RESULTS

Combining time-lapse with actin staining, collagen staining and phase contrast allowed us to follow the spatiotemporal progression of OC resorption events. Through the analysis of a total of five experiments (each with cells from a different human donor) involving 60 time-lapse recordings with a total duration of 3840 h we identified 299 independent resorption events made by 252 actively resorbing OCs. This gave us a substantial data set and allowed us to subcategorize the individual resorption events according to the way they were generated. It soon appeared that there were two different types of resorption modes, depending on whether the resorptive activity was mainly oriented parallel (like a bulldozer) or perpendicular to the bone surface (like a drilling machine), thereby leading to elongated or circular resorption cavities, respectively. These have been designated trenches and pits, respectively, according to our previously established terminology based on the shape of the excavations at the end of the cell culture (Merrild et al., 2015; Soe and Delaisse, 2010; Soe et al., 2013).

### Characterization of pit-forming OCs by time-lapse microscopy

**Fig. 1.**
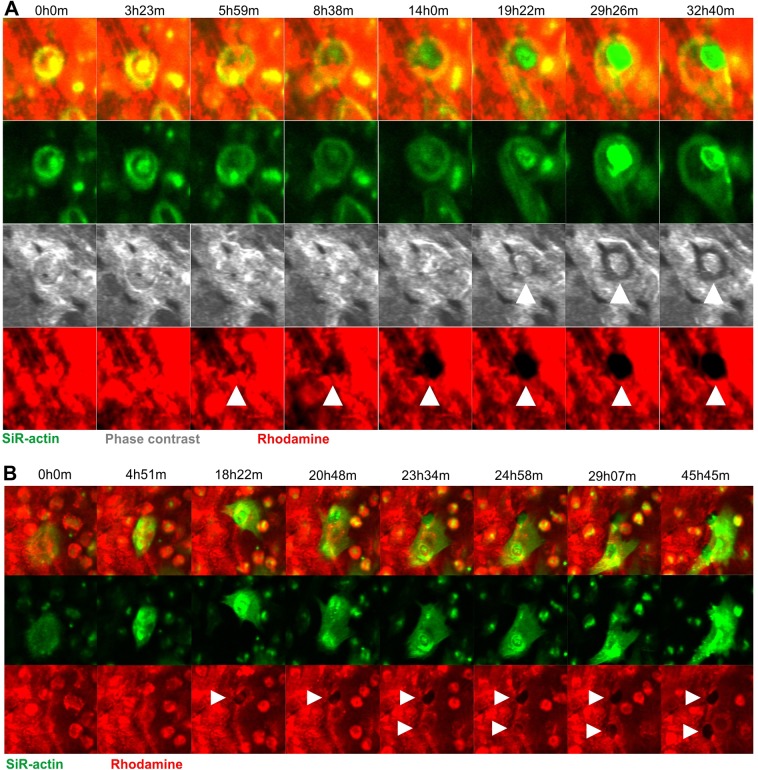
**Selected time-lapse images of OCs making pits.** Images were taken from Movies 1 and 2. (A) A selected time window out of a total recording time of 70 h showing an OC making a pit. Images were taken every 7 min. (B) A selected time window out of a total recording time of 70 h showing an OC making two pits. Images were taken every 21 min. White arrowheads point to the sites of resorption.

Analyses of the time-lapse recordings clearly identified that pit formation occurred exactly in line with the classical resorption cycle model: stationary resorption that is oriented perpendicular to the bone surface within the area delineated by the SZ. Resorption stops after some time, but may resume at another site after OC displacement, requiring the formation of a new SZ. Examples 1 and 2 of Movie 1 show two videos of pit formation, and Fig. 1A shows selected pictures from example 1 to illustrate some critical steps: the formation of a SZ reflecting OC activation (rows 1 and 2, 0h0m), clearance of collagen occurring centrifugally in accordance with collagen uptake at the center of the resorption compartment (Mulari et al., 2003) (row 4, 5h59m–4h0m), increased cavitation (row 3, 19h22m–29h26m) indicating that resorption is not limited to superficial collagen degradation, and increased presence of actin at the ruffled border area (row 2, 19h22m–29h26m). Note that the OC stands over the pit and that the SZ surrounds it during the whole resorption period, but the SZ seems to be very motile suggesting a continuous re-organization. Example 2 of Movie 1 confirms similar features during pit formation by another OC and shows particularly well the centrifugal clearance of collagen. It also illustrates the departure of the OC after resorption.

The video of Movie 2 highlights another aspect of the classical resorption cycle model: that the same OC can generate several distinct resorption events (pits) separated by OC migration or spreading, with the formation of a new SZ being required each time. Fig. 1B shows selected pictures of this movie to highlight critical steps: migration of an OC to a future resorption site (rows 1 and 2, 0h0m–4h51m), SZ formation and initiation of collagen removal (rows 1 and 3, 18h22m), spreading of the OC to the lower part of the picture (rows 1 and 2, 20h48m), formation of a new SZ and initiation of collagen removal at a new site (rows 1–3, 23h34m). Note that the SZs surround the respective pits during the whole period of their formation (rows 1 and 2), and are disassembled after these respective periods. Finally, resorption is terminated, the SZ disassembles and the OC moves away (rows 1 and 2, 29h07m–45h45m).

### Characterization of trench-forming OCs by time-lapse microscopy

**Fig. 2.**
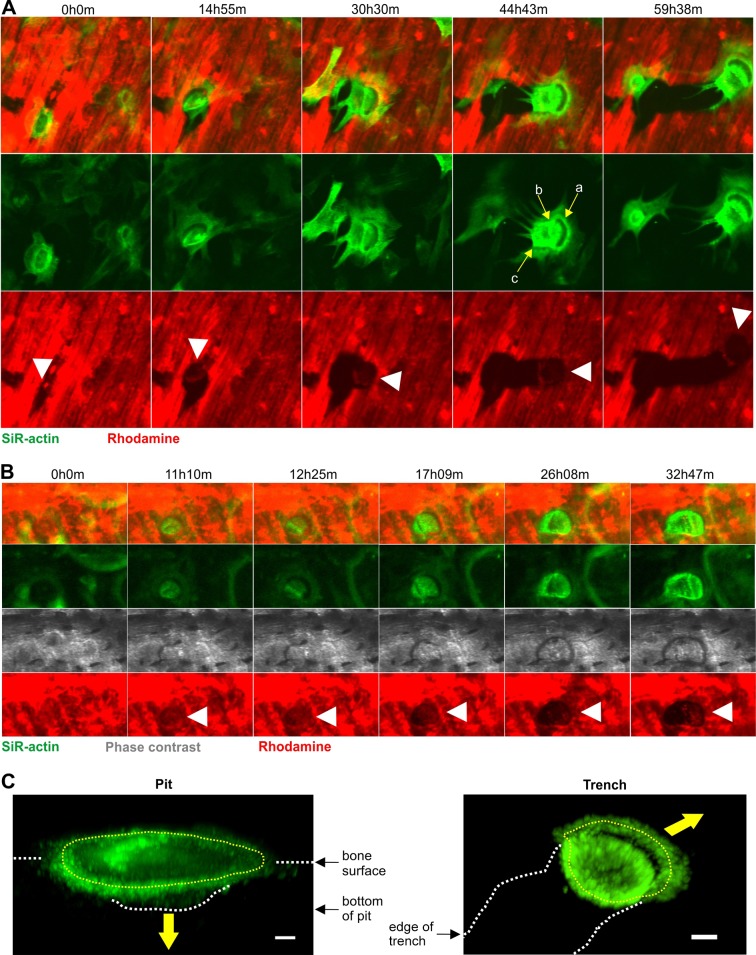
**Selected time-lapse images of OCs making trenches, and confocal images showing the 3D configuration of the SZ in pit- and trench-forming OCs.** Time-lapse images taken from Movies 3 and 4. (A) A selected time window out of a total recording time of 70 h showing an OC making a trench. Images were taken every 21 min. (B) A selected time window out of a total recording time of 70 h showing an OC starting out by making a pit and then transitioning into making a trench. Images were taken every 7 min. White arrowheads point to the sites of active resorption. Yellow arrows point to different actin zones corresponding to: a, the front-part of the SZ; b, the ruffled border and back-part of the SZ; c, the rear edge of the OC. (C) Pit- and trench-forming OCs were stained for actin by using phalloidin, and confocal pictures were taken. The illustrations show the respective 3D configurations of the SZs (dotted yellow line) and of the ruffled borders surrounded by these SZs in pit- and trench-forming OCs. From their orientations relative to the cavitation geometries, one can infer the directions of the resorption as perpendicular and parallel to the bone surface, respectively (yellow arrows). Both the pit- and trench-forming OCs are viewed at an angle of 45° to the bone surface. White dashed lines mark the zones of interest as indicated on the pictures. To improve the 3D understanding, Movies 5 and 6 show these images as rotating animations. Scale bars: 5 µm.

Analyses of our movies also identified many resorption events where the OC resorbed bone parallel to the surface for long periods (several days) and over long distances (several 100 µm) without any disassembly of the SZ. We have chosen to call them trenches in order to stress their continuous nature. Movie 3 shows three examples of trench formation. Overall these three examples show that trench-forming OCs move smoothly across the bone surface while simultaneously removing collagen. Importantly, there are no signs of interruption in this process, no matter whether images were made every 7 (example 1) or 21 min (examples 2 and 3). The SZ permanently appears as a crescent at the leading edge of the continuously expanding erosion (particularly visible in example 3). This SZ seems to be continuously displaced, and a disassembly/reformation of this SZ was not detected at any time point during the expansion of the trench. This shows the highly dynamic nature of the SZ and the continuous displacement of the resorption compartment in trench-forming OCs. Fig. 2A shows selected pictures of example 2 (Movie 3) stressing the characteristic long-distance erosion with the crescent-shaped SZ always visible at the leading edge.

### Most trenches are made by OCs that start in pit mode

A detailed analysis of OCs making trenches indicated that many actually start out in pit mode, and then transition to trench mode without disassembly/reassembly of the SZ. Movie 4 shows two examples documenting this mechanism, and Fig. 2B presents selected pictures from example 1 to illustrate some of the critical steps: the OC makes a SZ that remains stationary and surrounds an area of the bone surface (rows 1 and 2, 0h0m–12h25m) from where collagen is cleared (row 4, 0h0m–12h25m); ruffled border actin intensifies (row 2, 0h0m-12h25m) and a cavity becomes apparent (row 3, 11h10–17h09m) resulting in a pit; once a pit has been made the OC continues to resorb only on one side of the pit extending the cavity in one direction apparently without any disintegration of the actin ring (rows 1–4, 12h25m–32h47m); as collagen removal expands to one side, the SZ changes from an annular to a crescent configuration (row 2, 11h10m–17h09m). Note that the leading part of the SZ usually moves over the bone surface at a constant pace while the lagging part seems to be ‘pulled’ or drawn along (Movies 3 and 4). In order to understand the shape change of the SZ and its relationship with the change in orientation of the resorption axis, we visualized the 3D configuration of the SZ in pit- and trench-making OCs by using confocal microscopy (Fig. 2C; Movies 5 and 6). Interestingly, pit-forming OCs showed a SZ lying flat on the bone surface so that the ruffled border it surrounds is facing the floor of the excavation. This position indicates bone resorption perpendicular to the bone surface. In contrast, in trench-forming OCs only the leading edge of the SZ is on the bone surface and the rest adapts itself on the cavity walls so that the ruffled border is facing the front wall of the cavity. This position indicates mainly lateral resorption.

**Fig. 3.**
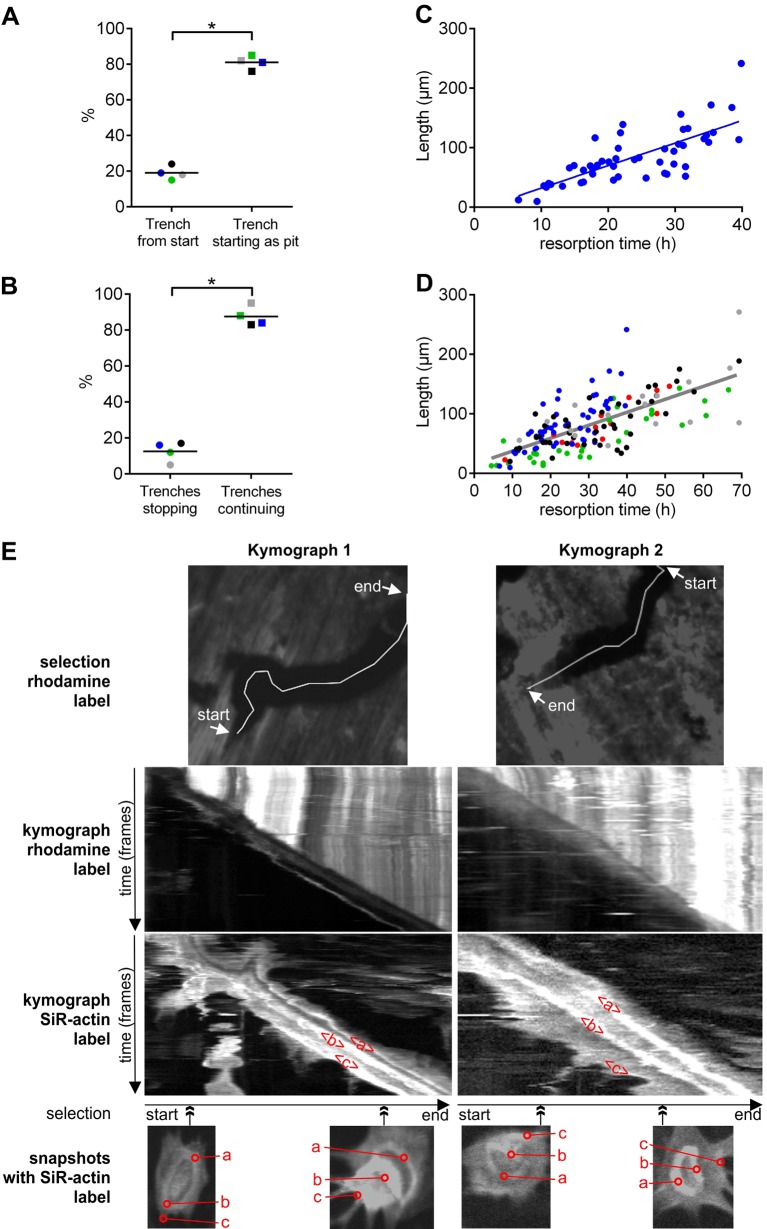
**Starting mode, duration/continuity and constant speed of trench-resorption events.** (A) The proportion of trench resorption events starting either as a pit or directly as a trench. These proportions were assessed in four different experiments, involving 33, 48, 34 and 17 events, respectively. (B) The proportion of OCs in trench mode that either stop or do not stop resorbing during the time-window of the time-lapse. These proportions were assessed in four different experiments, involving 33, 50, 36 and 22 events, respectively. The median proportions obtained in each experiment are shown as dots of a given color, the different colors refer to the respective experiments. Horizontal lines indicate the medians of these proportions. *P=0.029 (two-tailed Mann–Whitney test in both A and B). (C) Relationship between the length and time of resorption for all trenches identified in one single experiment (*n*=50). Linear regression gave a slope of 3.8 µm/h; r^2^=0.56, *P*<0.001 (Pearson correlation test). (D) A pool of all five experiments with all trenches identified (*n*=154), their respective lengths and the time it took to make them. A specific color indicates data from the same experiment. Linear regression gave a slope of 2.2 µm/h, r^2^=0.49, *P*<0.001 (Pearson correlation test). (E) Two representative kymographs monitoring Rhodamine and SiR-actin signals during trench formation: kymograph 1 is of the trench formation shown in Movie 3 (example 2) and Fig. 2A; kymograph 2 is of the trench formation shown in Movie 3 (example 3). The upper row of images show the respective selections of Rhodamine clearance. The middle two rows of images show the corresponding kymographs for the Rhodamine and SiR-actin label, respectively. The lower row of images shows snapshots of the F-actin organization of the OCs at the indicated point of trench formation (double-headed arrow). The actin zones ‘a’, ‘b’ and ‘c’ are as defined in Fig. 2A, and their respective displacements appear as correspondingly marked bands in the actin kymographs.

Quantifications of OCs making trenches in four experiments showed that ∼80% of them first made a pit and then extended this cavity into a trench (Fig. 3A). We were not able to get a clear picture of how the resorption of the other 20% started, but it appeared that collagen removal was initiated within a SZ that could take different shapes and sizes, and that collagen removal expanded only in one direction forming a trench from the beginning (data not shown).

### OCs in trench mode resorb for long periods of time without stopping, whereas OCs in pit mode show shorter periods of resorption

A remarkable property of trench-forming OCs is that once they initiated the trench, most continued extending it for the remaining duration of the time-lapse recording. We found that only ∼14% of the trench-forming OCs stopped during this period recording, and this percentage was reproducibly found in four different experiments (Fig. 3B). This long-duration resorption led to erosion over very long distances as shown in Fig 3C,D. Thus, the long trenches previously reported in the literature reflect long-duration resorption events. Fig. 3C shows that the time in which an OC was observed in trench mode correlates in a linear fashion with the length of the resulting trench (r^2^=0.56) (obtained from a single experiment). The slope of the curve indicates the mean resorption speed in µm/h, and, when looking at the speeds obtained across experiments, it is seen that the resorption speeds in trench mode does not vary notably between five experiments (2.1, 2.2, 2.2, 2.8 and 3.8 µm/h). When combining all data from these five experiments the resorption speed in trench mode is 2.2 µm/h (r^2^=0.49) (Fig. 3D). The constant speed of resorption all along the formation of individual trenches can be easily visualized on kymographs, as shown in Fig. 3E (these are the same events as shown in Fig. 2A and examples 2 and 3 of Movie 3). They stress that the removal of Rhodamine-labeled collagen and the displacement of the ruffled border (actin zone ‘b’ in Fig. 2A) occur at a constant rate and is not interrupted by migration/spreading events without resorption. The rear zone of actin (actin zone ‘c’ in Fig. 2A) follows with a variable lag, in accordance with the variations in OC spreading during the progress of trench formation (see Movie 3).

**Fig. 4.**
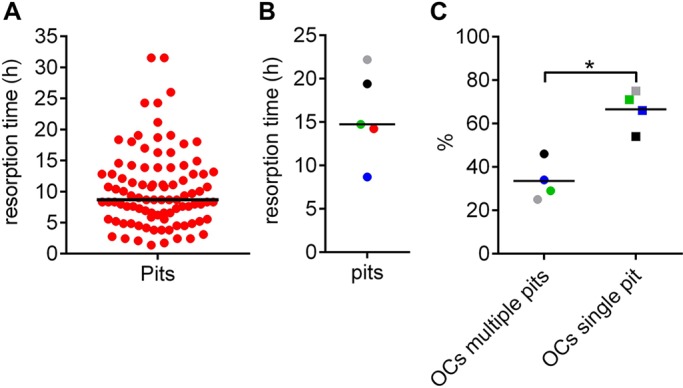
**Prevalence of multiple versus single pit-making OCs and duration of a pit resorption event.** (A) Duration of pit formation in a single experiment (*n*=94). Each dot represents the duration of a single resorption event. (B) Median durations obtained in five different experiments each represented by a dot. (C) The proportion of OCs that make either a single or multiple pits during the time-window of the time-lapse. These proportions were assessed in four different experiments, each involving 21, 38, 28 and 8 events, respectively. Horizontal lines indicate the medians of the prevalence. *P=0.029 (two-tailed Mann–Whitney test).

In contrast, the duration of pit formation was variable but shorter than that for trench formation. It ranged from 2 h to 32 h, with a median of 9 h in a single experiment (Fig. 4A), and the medians found in another four experiments ranged from 14 to 22 h (Fig. 4B). However, we also regularly observed that the same OC made several pits separated by periods of migration or spreading as shown in Movie 2 and Fig. 1B. A systematic evaluation of four different experiments showed that this was the case for 25 to 46% of the OCs – thus, roughly one third of them (Fig. 4C).

### OCs in trench mode erode the bone surface twice as fast as those in pit mode

**Fig. 5.**
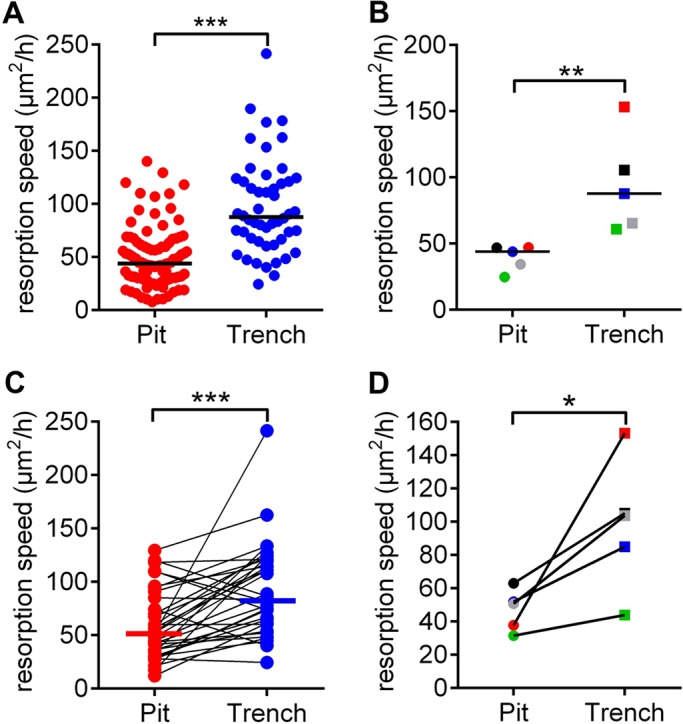
**Resorption speeds of OCs in pit and trench mode.** (A) The resorption events of a single experiment were classified as pits (*n*=94) or trenches (*n*=50). Each dot represents the speed of a resorption event. Horizontal lines indicate the medians. ***P<0.001 (two-tailed Mann–Whitney test). (B) Median resorption speeds of pit and trench formation, assessed in five different experiments (as shown in A) each represented by a dot. Horizontal lines indicate the medians. **P=0.008 (two-tailed Mann–Whitney test). (C) The respective resorption speeds that were recorded in the experiment shown in A are only shown here for individual OCs switching from pit to trench mode (paired results; *n*=34). Horizontal lines indicate the medians. ***P<0.001 (two-tailed Wilcoxon matched-pairs signed rank test). (D) Median speeds of five different experiments (as shown in C). *P=0.031 (one-tailed Wilcoxon matched-pairs signed rank test).

Fig. 5A shows the speed at which the eroded bone surface expands (µm^2^/h) for all pit and trench resorption events within a single experiment. A total of 94 pits (including those that transition into a trench) and 50 trenches were evaluated. First of all, it can be seen that there is a large variation in the resorption speeds within each category, but it is also highly significant that the median speed of trenches is twice that for pits. This finding is also reproducible and significant in five experiments (Fig. 5B). The speeds shown in Fig. 5A,B represent the median speeds of all resorption events, but since we found that trenches often start in pit mode and then switch into trench mode, it is particularly interesting to compare the resorption speed in these two modes for the same OC. Fig. 5C shows the paired analysis from a single experiment of the resorption speed of 33 OCs making a pit that transitions into a trench. Here, it is evident that the same OC almost always resorbs faster in trench mode than in pit mode. It is also interesting to observe that, although the resorption speed in pit mode is quite variable, it on average still doubles in trench mode. Across all five experiments, it is also evident that trench mode in general is significantly faster than pit mode for OCs making trenches (Fig. 5D).

### The resorption speed of an OC in pit mode varies depending on its subsequent resorptive activity

**Fig. 6.**
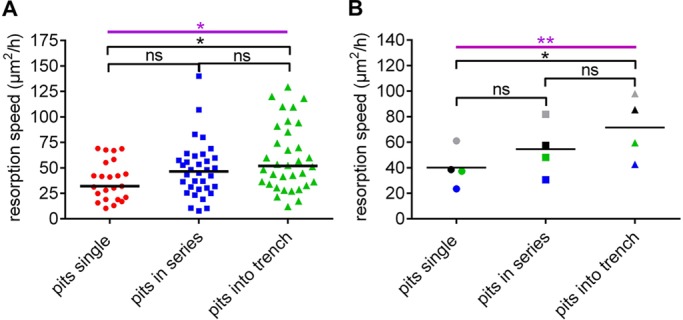
**The resorption speed of OCs in pit mode is related to their subsequent resorptive activity.** (A) OCs making pits within a single experiment were categorized according to how many and of which type of event they would make thereafter. The resorption speed of each OC within these categories were assessed and is shown as a dot. Horizontal lines indicate the medians of each of those categories. *P=0.027 (Kruskal–Wallis test; purple); *P=0.022; ns, not significant (left, *P*=0.390; right, *P*=0.575) (Dunn's multiple comparisons test; black). (B) Resorption speeds assessed in four different experiments for pits were categorized as in A. The median speeds obtained in each experiment are shown as dots of a given color, the different colors refer to the respective experiments. Horizontal lines indicate the medians of these speeds. **P=0.005 (Friedman test; purple); *P=0.014, ns, not significant (left, *P*=0.472; right=0.472) (Dunn's multiple comparisons test; black). Horizontal lines indicate the median values.

Since an OC in pit mode can stop resorbing, stop and then resume in pit mode elsewhere, or transition into the trench mode, we analyzed whether these different fates were related to the large variation of resorption speeds observed for pit mode (Fig. 5A). Fig. 6A shows that OCs making a pit that transitions into a trench resorb significantly faster than those only making a single pit. This difference in resorption speed between these categories could be repeated in four experiments (Fig. 6B). In this context, it is interesting to note that the areas of single pits and of those that precede a trench are not different (data not shown).

### Prevalence of OCs generating different types of resorption cavities

Finally, it is also important to assess the prevalence of these different types of resorptive behavior. Table 1 shows that out of 100 active OCs, 42 resorbed only in pit mode, 47 resorbed first in pit mode and switched thereafter to trench mode and 11 were in trench mode from the start. These numbers draw the attention to the importance of both the pit and trench resorption mode: the pit mode was used by 89% of the OCs either as single events or prior to trench mode, whereas the trench mode was used by 58% of the OCs either as the only resorption mode or the mode launched after initially being in pit mode. Note that these proportions relate strictly to the present situation and may change in other situations (Merrild et al., 2015; Panwar et al., 2016; Soe and Delaisse, 2010; Soe et al., 2013).

**Table 1.**
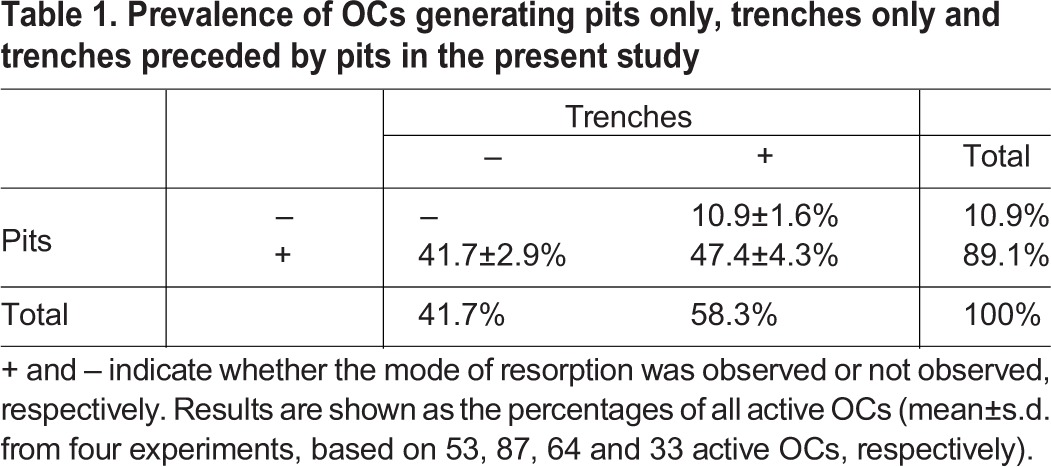
**Prevalence of OCs generating pits only, trenches only and trenches preceded by pits in the present study**

## DISCUSSION

**Fig. 7.**
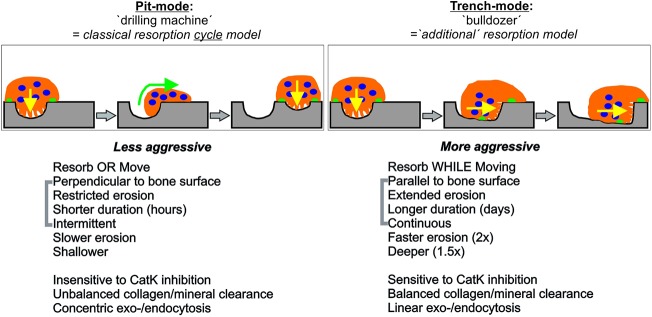
**Model showing the hallmarks of pit and trench resorption modes.** The pit mode is less aggressive and cyclical as it is interrupted to allow migration. In contrast, the trench mode is more aggressive and continuous, integrating resorption and migration in just one activity. See Discussion for more details.

It is commonly believed that resorption is achieved by stationary OCs drilling perpendicular to the bone surface, and that it alternates with periods of migration without resorptive activity (Georgess et al., 2014; Lakkakorpi and Vaananen, 1991; Novack and Faccio, 2011). This model greatly influences our current views about OC resorption, even if its universality has not been verified by direct observations. In our present study, ‘live’ monitoring of the spatiotemporal progression of resorption events shows that OCs resorb according to two distinct modes: one is in line with the widely accepted model of stationary resorption and is here called pit mode; the other is characterized by simultaneous resorption and displacement over the bone surface and is here called trench mode (Fig. 7). The latter mode is undertaken by more than half of the OCs investigated herein and shows several remarkable characteristics: long durations for a resorption event (days), spreading over long distances (several 100 µm) and high erosion speed (two times faster compared with pit mode). These findings indicate that the classical resorption model is not as universal as commonly thought, and that the trench mode deserves attention, as discussed below.

### The trench resorption mode allows the bone resorption compartment to move continuously over the bone surface

The key allowing us to determine that resorption and movement can happen simultaneously has been to monitor basic features of OC resorptive activity by time-lapse microscopy. This approach revealed continuous displacement of the SZ without any disruption, despite movement (Movie 3). Since the SZ surrounds the resorption compartment, this means that OCs in trench mode move over the bone surface while displacing their resorption compartment but without dismantling/rebuilding it at any time. This held true for all 154 OCs analyzed in trench mode. This lateral resorption is in accordance with the orientation of the ruffled border towards the front wall of the cavity (Fig. 2C) and not towards the floor of the cavity, as commonly shown for pit formation. This means that the polarization axes of resorption and migration coincide with each other and are parallel to the bone surface. Effective resorption was shown by concomitant collagen removal and cavity formation upon passage of the OC. Detailed analyses through kymographs stressed the very constant rate of collagen removal along with ruffled border displacement, and revealed neither interruption nor uncoupling of these activities. These observations held true even when increasing the chances of detecting possible interruptions by taking pictures every 7 min (i.e. a critical duration with respect with sealing zone turnover; see below) instead of every 21 min. Of note, our *in vitro* observations are in line with recent intravital time-lapse recordings visualizing the sub-osteoclastic resorption compartment itself (achieved with an acid-sensitive probe), and showing a crescent-shaped zone moving over the bone surface (Maeda et al., 2016).

Interestingly, the possibility that OCs can resorb and move at the same time has already been postulated, based on snapshot observations of OCs in culture (Lakkakorpi and Vaananen, 1991; Mulari et al., 2003; Stenbeck and Horton, 2000). Furthermore, phase-contrast videos of dentine-resorbing chick OCs performed in the Boyde laboratory drew attention to lateral resorption (Taylor et al., 1989). Observations of the SZ of these OCs highlighted that they do not appear as a planar ring lying on the intact bone surface, as usually illustrated, but show contact with the side and the bottom of the excavations in addition to contact with the native bone surface, thereby supporting an oblique orientation of the ruffled border and of resorption (Taylor et al., 1989) – which is confirmed in the present study. These authors stressed that this configuration of the SZ gives a crescent appearance when viewed from above – which is also the appearance of the SZ of trench-associated OCs reported by ourselves and others (Merrild et al., 2015; Mulari et al., 2003; Nesbitt and Horton, 1997; Rumpler et al., 2013; Stenbeck and Horton, 2000). Interestingly, previous quantifications of OCs with either a cresent- or an annular-shaped SZ (Mulari et al., 2003; Taylor et al., 1989) showed proportions very close to those we report herein. Of note, even histological observations of OCs involved in bone remodeling in their natural environment led to the idea that lateral resorption may be critical (Delaisse, 2014; Parfitt, 1993). Our present time-lapse data thus demonstrate the existence of a resorption mode that was already suspected in various studies, including ours (Merrild et al., 2015; Soe and Delaisse, 2010; Soe et al., 2013), but that remained hypothetical. These data allow us to conclude that many of the long trenches reported in the literature are not due to successive formation of confluent pits as regularly proposed (Georgess et al., 2014; Novack and Faccio, 2011; Rumpler et al., 2013; Vives et al., 2011), but to OCs that simultaneously resorb and move over the bone surface.

### Is a SZ compatible with movement?

The displacement of the SZ over the bone surface first appears counter intuitive, because the word ‘sealing’ suggests a fixed position. However, we feel that this displacement is in full accordance with the dynamic nature of the SZ – which is stressed in the literature and beautifully demonstrated in movies showing the displacement of GFP-stained actin rings over planar surfaces coated with mineralized collagen (Saltel et al., 2004). In fact, a close examination of movies of ‘stationary’ pit-forming OCs (e.g. Movie 1) already shows that the SZ is a bit unsteady even if it continues to surround the pit during the whole period of pit formation. This dynamic nature may be explained by the fact that the SZ consists of a superstructure of densely interconnected podosomes, which appear to have a median half-life of only 5 min (Luxenburg et al., 2012; Zhou et al., 2016). This short half-life thus renders continuous remodeling of the SZ mandatory. A coordinated formation and disintegration of podosomes at the inner and outer edge of the SZ would thus enable a unidirectional displacement of the seal while keeping an effective sealing in the center of the F-actin ring structure. This is reminiscent of how a tank chain (caterpillar tracks) always ensuring a tight contact zone despite a forward movement. The mechanism of this coordination is intriguing and remains to be investigated. Support for this view is found in the mechanism of expansion of podosome belts: new podosomes form at the outer edge while podosomes disintegrate at the inner edge (Tehrani et al., 2006).

### Specific characteristics of trench-forming osteoclasts

At a first glance it may appear as if pit and trench mode are unrelated. But detailed analyses showed that 81% of all OCs making trenches actually started in pit mode and then transitioned into the trench mode without any detectable delay or disintegration and rebuilding of the SZ. Thus, the pit mode remains critical even for generation of trenches, and 90% of all active OCs undertake pit mode at least to initiate resorption. The present ‘live’ observations of transition from pit to trench mode fully support the model of trench generation we proposed in our earlier reports (Soe and Delaisse, 2010; Soe et al., 2013). This model was based on a series of data showing that the rate of collagenolysis relative to that of demineralization must be high to allow OCs to switch from pit to trench mode (Soe et al., 2013). A major finding among these data was that inhibition of cathepsin K completely abolished trenches while simultaneously enhancing pits (Panwar et al., 2016; Soe et al., 2013). Another critical observation is that there is a greater removal of collagen from trenches compared to from pits, as evaluated by scanning electron microscopy and X-ray spectroscopy (Panwar et al., 2016; Soe and Delaisse, 2010). Based on these results, one may envision distinct subtypes of OCs that differ in their collagenolytic potential and thereby also differ in their ability to switch from pit to trench mode. The data shown in Fig. 6 support this view. They show that OCs that only make a single pit resorb slower than those that shift into trench mode after making the initial pit. Thus, the resorption machinery of trench-forming OCs already appears more effective in the initial pit mode, compared to that of OCs that cannot make a trench. However, more work has to be done to fully understand this mechanism and one can think of different working hypotheses relating collagen degradation to trench formation: (1) that if collagen is not degraded/cleared fast enough, it accumulates and acts as an antagonist of the polarized resorption phenotype of the OC (Saltel et al., 2004; Takahashi et al., 2007), thus resorption stops at the pit stage; (2) that small collagen fragments may be necessary for feeding an endocytic recycling pathway, since such pathways are known to be involved in polarized cell migration (Jones et al., 2006); or (3) that the quality of collagen degradation/clearance may affect the sharpness of edges of resorption cavities, and nanotopography is believed to influence the SZ stability (Geblinger et al., 2010) and may affect cell guidance (Sun et al., 2015). Note that collagen degradation is not the only important aspect of these hypotheses: its clearance is also important to consider, and in relation to clearance, it is of interest that the arrangement of exocytosis and endocytosis at the ruffled border appears to be linear in the trench mode while it is concentric in pit mode (Mulari et al., 2003).

### Aggressiveness of trench-making osteoclasts and clinical relevance

We have previously suggested that trenches reflect a more aggressive form of resorption than pits because we, for example, found that trenches are deeper than pits (Merrild et al., 2015). Our present time-lapse study allowed us to quantify even more characteristics indicating higher aggressiveness: OCs in trench mode can resorb for days (at least 69 h) while OCs in pit mode stop after a median of 13 h; trenches constantly get longer with time and reached a median length of 76 µm, while pits only reached a median diameter of 19 µm during our recording; the linear rate of erosion was on average 2.2 µm/h in trench mode but only 0.8 µm/h in pit mode. Note that these values are of the same order of magnitude as the 1.8 µm/h reported for the elongation rate of Haversian canals in canine ribs (Jaworski et al., 1975), but well below the rates of pure migration (median of 97 µm/h) reported for rabbit OCs on bone slices (Kanehisa and Heersche, 1988). We also show that the higher erosion rate in trench mode is a specific property of this resorption mode and not only related to a possible subtype of OC, since the same OC roughly doubles its erosion speed when switching from pit mode into trench mode (Fig. 5C). The clinical relevance of this higher aggressiveness should be considered in the context of several results reported previously: that trenches and pits have been demonstrated *in vivo* (Boyde et al., 1986; Dempster et al., 1986; Gentzsch et al., 2003; Jones et al., 1985; Merrild et al., 2015); that trenches make bone more fragile than a matching level of erosion due to pits (Vanderoost et al., 2013); that the ability of OCs to make trenches varies considerably depending on the blood donor they were generated from (Merrild et al., 2015); and that drugs such as glucocorticoids enhance the proportion of eroded surface by facilitating more trenches (Soe and Delaisse, 2010), whereas drugs such as odanacatib decrease it (Panwar et al., 2016). As discussed elsewhere (Soe et al., 2013), the cells of the OC microenvironment are expected to influence the selection of the resorption mode. Thus, pits and trenches are not simply an intellectual curiosity. The severity of OC resorption should not be merely evaluated through measuring the extent of erosion, but should also discriminate between erosion generated by OCs in trench or pit mode.

### Conclusion

We conclude that OCs can integrate resorption and migration in just one activity (like a bulldozer), leading to ‘trenches’ (Fig. 7). This trench resorption mode enables high aggressiveness, and is undertaken by more than than half of the active OCs in the present study. The integration of resorption and migration in just one activity provides a new model explaining how an OC moves its resorptive activity over the bone surface. It is in marked contrast with the widely accepted bone resorption cycle model, that is based on resorption events exerted perpendicular to the bone surface by stationary OCs, and that alternate with cell migration/spreading episodes, therefore leading to a series of pits (like a drilling machine). This resorption cycle model was considered so far as the only way to explain how OCs move their resorption activity over the bone surfaces. Beyond bone resorption, the paradoxical integration of resorption and migration by a trench-forming OC, highlights that a cell can simultaneously exhibit epithelial and mesenchymal characteristics. To our knowledge, this is a unique situation that deserves attention in future research.

## MATERIALS AND METHODS

### OC preparation

CD14^+^ monocytes were isolated from blood of human donors (approved by the local ethical committee, 2007–2019, with written consent obtained from each donor) by centrifugation through Ficoll-Paque (Amersham, GE Healthcare, Little Chalfont, UK), and subsequently suspended in 0.5% bovine serum albumin (BSA) and 2 mM EDTA in PBS, and were purified by using BD IMag™ Anti-Human CD14 Magnetic Particles - DM (BD Biosciences, CA) according to the supplier's instructions (Moller et al., 2017). CD14^+^ cells were seeded at a density of 66,667 cells/cm^2^ in T75 culture flasks (Greiner, InVitro, Fredensborg, Denmark) supplied with α minimum essential medium (αMEM; Invitrogen, Taastrup, Denmark) containing 10% fetal calf serum (FCS; Sigma-Aldrich, St Louis, MO) and 25 ng/ml human macrophage colony-stimulating factor (M-CSF; R&D Systems, Abingdon, UK) and cultured for 2 days at 37°C in 5% CO_2_ in a humidified atmosphere (Soe and Delaisse, 2010). Floating cells were harvested by centrifugation (500 ***g*** for 5 min) and returned to the respective flasks in fresh αMEM with 10% FCS, 25 ng/ml human M-CSF and 25 ng/ml human RANKL (also known as TNFSF11). The cells were cultured for an additional 7 days with medium changed twice.

### Time-lapse recordings

Cortical bovine bone slices with a thickness of 0.4 mm (BoneSlices.com, Jelling, Denmark) were labeled with N-hydroxysuccinimide ester-activated Rhodamine fluorescent dye (ThermoFisher Scientific, Waltham, MA, USA) as described (Mulari et al., 2003). Analyses of confocal images showed that this technique labeled protein or collagen down to a depth of 5.7±0.8 µm (±s.d.) below the bone surface. Matured OCs were lifted with accutase, harvested by centrifugation (500 ***g*** for 5 min) and resuspended in αMEM with 10% FCS, 25 ng/ml M-CSF and 25 ng/ml RANKL. Cells were seeded at a density of 100,000 cells per bone slice in a 96-well plate. In order to label F-actin in living OCs, 100 nM SiR-actin (excitation at 652 nm; emission at 674 nm) and 10 µM verapamil (both supplied by Spirochrome, Stein am Rhein, Switzerland) were added and incubated for 5 h at 37°C in 5% CO_2_ in a humidified atmosphere. Subsequently, bone slices were transferred to wells of Nunc Lab-Tek II chambered cover-glass (ThermoFisher Scientific) in medium containing M-CSF, RANKL, SiR-actin and verapamil as described above. Time-lapse images were made using an Olympus Fluoview FV10i microscope (Olympus Corporation, Shinjuku, Tokyo, Japan) at 5% CO_2_ and 37°C, with a 10× objective with a confocal aperture of 2.0 corresponding to a *z*-plane depth of 21.2 µm. The initial focus was set on the bone surface. Recordings were made for a period of 40 to 70 h taking images every 7 or 21 min (four or 12 recording zones, respectively). Most commonly, surface areas between 0.3 to 0.4 mm^2^ were recorded in each zone. Verapamil was used because our initial tests showed that the quality of the SiR-actin staining was improved in its presence. Neither SiR-actin nor verapamil affected the extent of resorption, the proportions between pits and trenches or the length of trenches (data not shown).

### Analyses of time-lapse recordings

Intensities of each image series and channels were optimized by using the Olympus Fluoview 4.2 Viewer (Olympus Corporation) and were exported in .avi format. These movies were further analyzed with ImageJ 1.50f (National Institutes of Health, USA) with respect to length and area measurements in pixels. These were subsequently converted into micrometers by using the information from the Olympus Fluoview 4.2 Viewer data-manager. The duration of each event was estimated by counting the number of frames from beginning to end of the particular event. Since there were either 7.0 or 20.8 min between each frame these could be converted into hours and minutes. Since the Rhodamine labeling penetrated 5.7±0.8 µm into the bone, a complete removal of Rhodamine dye meant that collagenolysis had reached a depth of at least 4.9 µm. Pits were identified based on OCs that only displayed a round actin ring and that did not move the ring during resorption. This could be clearly identified when comparing the movies using the different channels. The start of a pit resorption event was determined by the first appearance of an F-actin staining in the center of the actin ring (formation of the ruffled border) along with the first signs of centrifugal collagen removal of the bone surface staining. The end of the resorption event was determined by the disappearance of ruffled border, the lack of signs of collagen removal and/or the OC migrating away from the event. Pits were furthermore categorized into single or multiple based on whether the same OC made only one or more pits during the time of recording. Trenches starting as a pit were identified as follows: an initial formation of a pit was identified by a clear formation of a circular stationary actin ring and by a centrifugal removal of collagen. Termination of the pit mode and transition into trench mode was determined by the first sign of movement of the actin ring towards one side of the pit (beginning to form the crescent-shaped actin ring) and of collagen removal on this side. Resorption starting as a trench was used to categorize an event that did not start by removing the collagen centrifugally, but instead initiated collagen removal on a ‘line’ that expanded only in one direction, forming a trench and having a crescent-shaped actin ring. The areas of all these events were determined by manually tracing the edges of the resulting resorption cavities appearing as ‘black’ areas on the Rhodamine staining. In the case of ‘trenches starting as pits’, the area of the initial pit was analyzed separately and the area of this pit was subtracted from the total area of the resulting trench. For length measurements, a free-hand line was placed in the center of the trench following the trail of the resorbing OC. It was noted whether an OC making a trench stopped resorbing during the recording or whether they were on-going when the recording stopped. Trenches that appeared in the frame during recording were only used to calculate general resorption speeds, but were not categorized according to their start since this was not possible. Pits that had started before the recording started were only categorized, but their resorption speed was not determined. If this pit transformed into a trench the resulting trench was analyzed and categorized accordingly.

### Kymograph analysis

In total, time-lapse recordings of 13 trenches were analyzed by making kymographs of both the Rhodamine and SiR-actin label, in order to show their changes in time and space. The analysis was performed with ImageJ 1.50f.

### Confocal microscopy

Staining of F-actin in fixed OCs on bone slices and confocal microscopy were performed as described previously (Merrild et al., 2015). Images were processed using Imaris version 7.6.5 (Bitplan AG, Zurich, Switzerland) to highlight the 3D conformations of the SZ. Movies of the rotating 3D images were generated using the Imaris software.

### Statistics

All graphs and statistical analyses were performed using Graph Pad Prism version 6.07. The statistical tests used are indicated in the figure legends along with the indication of the exact *P*-values. When analyses were performed for all experiments, it is the median values from each experiment that are shown.
